# Tracking Multiple Vehicles Constrained to a Road Network From a UAV with Sparse Visual Measurements

**DOI:** 10.3389/frobt.2021.744185

**Published:** 2021-10-21

**Authors:** Jared J. Moore, Craig C. Bidstrup, Cameron K. Peterson, Randal W. Beard

**Affiliations:** Department of Electrical and Computer Engineering, Brigham Young University, Provo, UT, United States

**Keywords:** cooperative control, unmanned air vehicles, multiple target tracking, computer vision, particle filter

## Abstract

Multiple-target tracking algorithms generally operate in the local frame of the sensor and have difficulty with track reallocation when targets move in and out of the sensor field-of-view. This poses a problem when an unmanned aerial vehicle (UAV) is tracking multiple ground targets on a road network larger than its field-of-view. To address this problem, we propose a Rao-Blackwellized Particle Filter (RBPF) to maintain individual target tracks and to perform probabilistic data association when the targets are constrained to a road network. This is particularly useful when a target leaves and then re-enters the UAV’s field-of-view. The RBPF is structured as a particle filter of particle filters. The top level filter handles data association and each of its particles maintains a bank of particle filters to handle target tracking. The tracking particle filters incorporate both positive and negative information when a measurement is received. We implement two path planning controllers, receding horizon and deep reinforcement learning, and compare their ability to improve the certainty for multiple target location estimates. The controllers prioritize paths that reduce each target’s entropy. In addition, we develop an algorithm that computes the upper bound on the filter’s performance, thus facilitating an estimate of the number of UAVs needed to achieve a desired performance threshold.

## 1 Introduction

Multiple-target tracking has a wide array of applications ranging from air traffic control (Li and Bar-Shalom (1993)) to following shoppers in a store (Liu et al. (2007)). Many approaches exist to track moving objects, vehicles, and pedestrians. Algorithms of particular interest include Multiple Hypothesis Tracking (MHT) (Reid (1979)), Probability Hypothesis Density (PHD) filters (Clark et al. (2007)), Recursive RANSAC (R-RANSAC) (Niedfeldt and Beard (2014)), and their variants. Most applications of these algorithms constrain the area of interest to the field-of-view of the sensor deployed. Targets that move out of the field-of-view are usually forgotten and considered a new target when seen again. Other research, where the area of regard is larger than the field-of-view of a UAV’s sensor, sometimes pose the situation as a search problem, as in (Allik (2017)) and (Wong et al. (2005)).

Another common strategy is to use cooperating UAVs to increase coverage beyond the single sensors field-of-view (e.g. (Yuan et al. (2018)), (Wolek et al. (2020)), and (Haoran et al. (2020))). While this approach is advantageous and has generated many novel control and estimation approaches, there is a lack of specification on how additional UAVs will affect the tracking capabilities. This work provides that connection between the number of UAVs operating in a region with the resulting tracking fidelity. In this way trade-offs between the costs of deploying additional UAVs may be evaluated against the required tracking performance.

Target tracking may also be improved by considering features in the operational environmental. It was shown in (Cheng and Singh (2007)) and (Booth et al. (2020)) that incorporating road network information, improves the tracking capabilities. In (Ding et al. (2017)) additional domain knowledge (e.g. traffic rules and effects of neighboring vehicles) was combined with a moving horizon estimator and multiple hypothesis tracker to further improve performance. These approaches require that the domain information be known a priori. However, as was shown in (Moon and Peterson (2018)), incorporating road network information is possible using open source products such as OpenStreetMap (Haklay and Weber (2008)).

Another powerful technique, employed by (Allik (2017)) and (Ahmed et al. (2017)), is to incorporate negative information. Traditional localization would only update the target location belief if it were viewed. However, if the target is not detected within the UAV’s field-of-view, this still gives some information about its location. As an illustration, consider the case where a target could be in one of two possible locations. Searching one location will reveal that the target is indeed there or must be at the other location. This negative information update proves valuable when a UAV cannot follow all of the targets all of the time.

There has been extensive research aimed at improving the quality of target tracks. This research includes, optimizing sensor-to-target viewing geometries (He et al. (2020); Peterson (2017)), improving the quality of the sensed data (Oktay et al. (2018)), and providing control techniques to reduce the error between planned and realized UAV paths (Farrell et al. (2019)). These specific techniques fall outside the scope of this work. However, they could be layered on top of this research to further enhance the UAV’s tracking performance.

In this paper, we consider the task of performing surveillance on multiple targets with a single UAV in a rural, hilly area. The operators want to have a good understanding on where the targets visit over a long time span, but the terrain or distance between roads makes it difficult to observe the targets without flying over the roadway, even with a gimballed camera. An even more difficult environment would be an urban canyon, where an altitude-constrained UAV could only see along corridors.

We develop a method for incorporating road map information as well as a negative update to track multiple vehicles in an area larger than the UAV’s sensing field-of-view in the presence of clutter and missed detections. The road map information provides a constraint on the allowable vehicle paths, this restricts the UAV’s search space and enables the UAV to re-discover vehicles when there is temporal sparsity between vehicle sightings. The negative updates ensure that the absence of vehicle sightings gets incorporated into the estimated target position. We assume it is important to label and properly distinguish between targets. However, we assume that sensors are not capable of classifying them (e.g. if the radar cross sections are similar or if using a camera, the number of pixels on target is too low for differentiating targets). We use simulations and hardware experiments to show that our target tracking approach is successful despite these challenging restrictions.

In addition, this paper develops a novel method for maintaining target certainty by the tracker. In particular, we identify the particle filter’s average-entropy lower-bound using the size and complexity of the map, the number of targets tracked, and the number of active UAVs. The lower bound on entropy is inversely proportional to the upper bound on the target location certainty. By knowing the upper bound on the target location certainty for a given number of UAVs, we can predict the number of UAVs needed to achieve a defined performance threshold for any given scenario. To the best of our knowledge no other work has developed an approach to address this issue.

The Rao-Blackwellized particle filter (RBPF) and the receding horizon path planner were previously described in (Bidstrup et al. (2019)). The current paper expands on those results, providing novel contributions that include *1*) the application of the RBPF with negative update information to tracking multiple targets along road networks, *2*) a theorem for computing the particle filter’s lower bound of the average entropy, and *3*) path planning algorithms, including a new neural net path planner trained with deep reinforcement learning (deep-RL), *4*) extensive simulation and hardware experiments of end-to-end framework.

The UAV motion is controlled utilizing the road network constraint and the particle filter, which provides probability density information about the predicted target location. Numerical simulations demonstrate the comparable effectiveness between different controllers and show that the controllers improve target estimate certainty when compared to a random search pattern. Hardware results completed in a motion capture room show the efficacy of an end-to-end solution.

The remainder of the paper is organized as follows: Section 2 describes a particle filter for tracking a single target. Section 3 extends the method to multiple targets with unknown data association by creating a high level particle filter where each particle is a single-target particle filter as described in Section 2. The receding horizon controller (RHC) and deep-RL controller presented in Section 4 leverage the particle filter output. Section 5 derives an algorithm to compute the lower bound on the average-entropy of the overall scheme. Results comparing the two controllers to a random search pattern are presented in Section 6. Hardware results showing the RHC integrated with the particle filter are presented in Section 7. Finally, conclusions are given in Section 8.

## 2 Target Tracking

Consider the problem of tracking a single vehicle driving along a road network with a UAV flying overhead. We are assuming that the UAV has a limited field-of-view (FOV) and can only see the roadway beneath it (e.g. through a downward pointing camera). This is a reasonable approximation of real-world scenarios such as operating in an urban canyon, hilly terrain, or over a large expanse where even a gimbaled camera could not resolve targets on distant roads. The solution described in this section is used as a building block in the complete architecture of tracking multiple vehicles with unknown data correspondence presented in Section 3.

### 2.1 Single-Target Particle Filter

**FIGURE 1:**
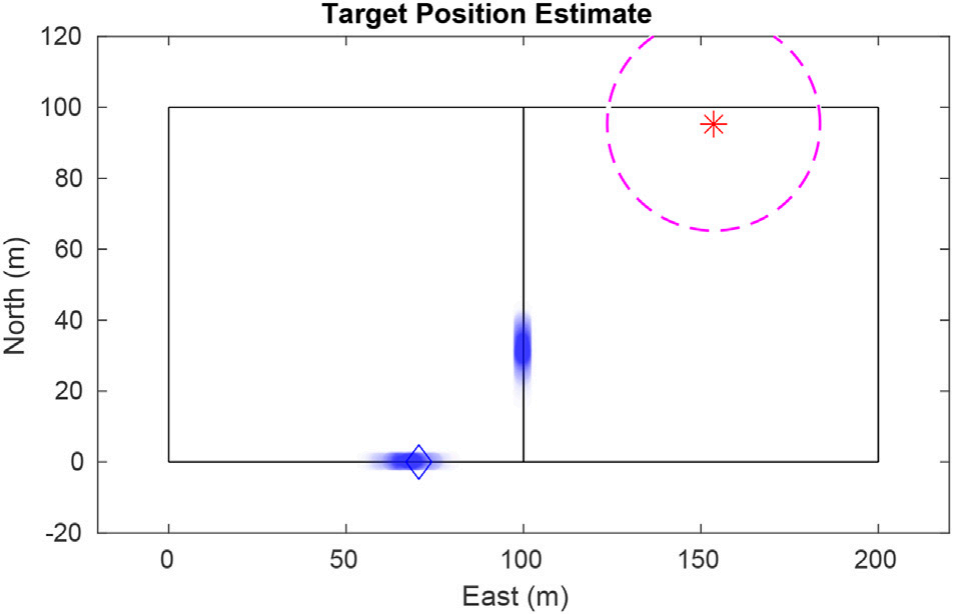
The single-target particle filter maintains a finite number of hypotheses, even after the vehicle has traveled some distance since being seen. Particles are plotted as transparent dots to indicate density. The blue diamond shows true target position, the star shows the UAV position, and the dashed circle delineates the UAV’s field-of-view.

The UAV encodes its belief of the target location using a particle filter. Also known as Sequential Monte Carlo, the particle filter is a nonparametric implementation of the Bayes filter (Arulampalam et al. (2002)). In contrast to a Kalman filter, the particle filter easily describes multimodal distributions, cleanly handles nonlinear motion and measurement models, and allows for mixed continuous-discrete state representations. These features are especially helpful in our scenario, where a target vehicle could be any distance along (continuous) any one of a number of roads (discrete) after passing through an intersection. Figure 1 illustrates this scenario where the UAV has not seen the target for some time, and multiple good hypotheses exist.

Let *x* denote the state of the target, which includes the current position, as defined by its current edge and position along that edge, as well as the speed it is travelling. We encode the initial belief of the state as a probability density function (pdf) *p*(*x*_0_) and draw the initial set of particles $X_{0}$ from this distribution,

$$X_{0}∼p\left(x_{0}\right).$$ (1)

Each particle is denoted as $x_{k}^{n}$ where the superscript *n* denotes the *n*th particle and the subscript *k* denotes time index. The set of particles at time *k* is denoted as $X_{k}=\left\{x_{k}^{n}|n=1\ldots N\right\}$ . Particles contain all the information present in the target state, but also have a belief value representing the certainty that a particle represents an actual target. In this paper we chose a uniform distribution for *p*(*x*_0_), implying no prior knowledge about where the target may initially be in the search area. Prediction is performed by sampling from the proposed distribution as

$$x_{k}^{n}∼p\left(x_{k}|x_{k-1}^{n}\right).$$ (2)

When a measurement *y*_*k*_ is received, each particle is assigned an importance factor as the ratio of the target distribution to the proposed distribution

$$w_{k}^{n}=\frac{p\left(x_{k}^{n}|y_{1:k}\right)}{p\left(x_{k}^{n}|y_{1:k-1}\right)},$$ (3)

Where *y*_1:*k*_ = {*y*_1_, …, *y*_*k*_} is the set of measurements. By applying Bayes’ rule to the numerator and factoring, we see that the importance factor, or weight, is proportional to the likelihood of the current measurement, given the particle’s current state:

$$w_{k}^{n}\propto p\left(y_{k}|x_{k}^{n}\right).$$ (4)

With the added constraint that all weights must sum to one, the proportionality is sufficient to calculate each particle’s weight. At each time instant, the particles are resampled with probability proportional to their weights, and their weights are reset to the initial value $p_{0}=\frac{1}{N}$

We employ two techniques to better fit the posterior distribution *p*(*x*_*k*_ ∣ *y*_1:*k*_). First is the low variance resampling technique described in (Thrun et al. (2006)). While resampling is necessary it can remove good particles and lead to particle deprivation. Low variance resampling helps mitigate this issue. Another technique is to resample only as often as is beneficial, known as selective resampling (Grisetti et al. (2005)). The idea behind selective resampling is that if the particles were sampled from the true posterior, they would all have equal importance. The deviation from the true posterior can then be estimated by calculating the number of effective particles, a metric given by (Liu (1996)) as

$$N_{eff}=\frac{1}{\sum_{n=1}^{N}{\left(w^{n}\right)}^{2}}.$$ (5)

Selective Resampling provides a way to determine when resampling is necessary. For example, the particles could be resampled when *N*_*eff*_ drops below the threshold $\frac{2N}{3}$ To calculate *N*_*eff*_, a particle must keep track of its importance factor through each measurement update until resampling occurs, i.e.,

$$w_{k}^{n}=\eta_{w}w_{k-1}^{n}p\left(y_{k}|x_{k}^{n}\right),$$ (6)

Where *η*_*w*_ is a normalizing factor for the particle weights. In practice, these two techniques greatly reduce the chance that good particles are lost during resampling.

### 2.2 Road Constraint

Let F(x) denote the field-of-view of the camera in the inertial frame when the UAV is at state *x*. Any time a target is outside the UAV’s field-of-view, its state can only be estimated using prediction. If the target could move unconstrained on the ground plane, the estimate would quickly disperse and become unusable. Constraining the target to a road network allows the UAV to accurately predict the possible places the target could go, even when it has not been seen for some time (*see* Figure 1).

We model the road network constraint as a directed graph

G=(N,E), (7)

Where edges E represent road segments, and nodes N represent intersections or corners with known Cartesian coordinates. In the remainder of this paper we will not distinguish between the graph and its embedding in $R^{2}$ , i.e., n∈N will represent a point in $R^{2}$ representing the inertial north-east coordinates of the node, and e∈E represents an inertially defined line in $R^{2}$ with length len(*e*). Each particle encodes its current edge *e* and how far along the edge it has traveled, denoted by path parameter *s*. Therefore the *nth* particle is given by,

$$x^{n}=\left(e^{n},s^{n}\right),\;e^{n}\in E,\;s^{n}\in R.$$ (8)

We use the notation $G\left(x^{n}\right)$ to denote the real-world 2D Cartesian location associated with particle *x* ^*n*^.

### 2.3 Target Motion Model

The dynamic model of the target motion defines the proposed distribution shown in Eq. 2. While virtually any dynamic model works with this architecture, this paper uses a constant velocity model with random perturbations. The particle’s position *s* ^*n*^ is propagated along the road as

$${\dot{s}}^{n}=v_{e^{n}0}+\nu ,$$ (9)

Where $v_{e^{n}0}$ is some nominal velocity for the road segment *e* ^*n*^, (e.g., a posted speed limit of 15 m/s) and $\nu ∼N\left(0,\sigma_{\nu }^{2}\right)$ is additive Gaussian white noise with standard deviation *σ*_*ν*_.

When a particle reaches an intersection (i.e., the end of an edge), in other words, if *s* ^*n*^ > ‖*e* ^*n*^‖, then *e* ^*n*^ is randomly assigned with equal probability to one of the edges leaving that node, excluding the edge that returns to the previous node (i.e., no U-turns).

### 2.4 Measurement Model

When a sensor measurement is detected, the measurement likelihood model is a mixture of a Gaussian distribution corresponding to a true measurement coming from a target that is in the camera field-of-view F and a uniform distribution corresponding to a false alarm, specifically,

$$p\left(y_{k}|x_{k}^{n}\right)=\left(1-P_{\text{FA}}\right)\frac{1}{2\pi \sigma_{\tau }}\exp\left(-\frac{1}{2\sigma_{\tau }}\|G\left(x_{k}^{n}\right)-y_{k}{\|}^{2}\right)+\frac{P_{\text{FA}}}{A_{R}},$$ (10)

Where *P*_FA_ is the probability of false alarm, *σ*_*τ*_ is the standard deviation of the measurement noise, and *A*_*R*_ is the 2D area spanned by the road network.

### 2.5 Negative Update

The lack of a sensor measurement is also information that can be used to update the target likelihood map because it indicates that the target is either not in the sensor field-of-view F or the detection was missed by some probability of false-negative *P*_FN_. In this case, the negative measurement model is a mixture of two uniform distributions, The negative measurement model is then a mixture of two uniform distributions,

$$p\left(z_{k}|x_{k}^{n}\right)=\left\{\begin{array}{cc} \frac{1-P_{\text{FN}}}{A_{F}},\;\; & G\left(x_{k}^{n}\right)\in F\\ \frac{P_{\text{FN}}}{A_{R}-A_{F}},\; & \text{Otherwise} \end{array}\right.$$ (11)

Where $A_{F}$ is the area of the UAV’s field-of-view. When using a camera fixed with respect to the UAV body frame, F and consequently $A_{F}$ , become a function of the UAV altitude and attitude. For the purposes of this paper, we assume a constant *P*_*FN*_.

## 3 Data Association

Section 2 describes tracking a single target in the presence of clutter and missed detections. In this section, we extend the filter to track multiple vehicles. Subsection 3.1 describes the tracking problem with perfect target identification, we then extend that in Subsection 3.2 to stochastically handle unknown data correspondence.

### 3.1 Known Data Correspondence

Extending single-target tracking to multiple targets would be trivial if sensor measurements could give perfect data correspondence. That is if the sensor reported both the location and ID of the target. We assume that each target’s motion is independent of other targets, implying that the joint distribution can be factored as

$$p\left(X_{k}^{1:M}|y_{1:k}\right)=\overset{M}{\underset{m=1}{\prod }}p\left(X_{k}^{m}|y_{1:k}\right),$$ (12)

Where *M* is the number of targets to be tracked, and $X^{m}$ is the set of particles estimating the location of the *m* ^th^ target. In the case of perfect data correspondence the UAV simply maintains a separate particle filter for each target. As a positive measurement is received, it would only be applied to the corresponding target. Negative measurements would be applied to the entire bank of trackers.

Unfortunately, it can be very difficult to visually differentiate two similar looking vehicles and so perfect data correspondence is not possible. Instead, we implement a Rao-Blackwellized Particle Filter (RBPF) to handle the data association in a manner similar to (Ahmed et al. (2017)) and (Särkkä et al. (2007)).

### 3.2 Rao-Blackwellized Particle Filter

Let *c*_1:*k*_ be the history of data associations; that is, *c*_*k*_ = *m* indicates that the measurement at time *k* corresponds to target *m*, where *m* ∈ 1…*M* and *M* is the number of targets. If we let *c*_*k*_ be a random variable, then the joint distribution given a certain measurement is

$$p\left(c_{1:k},X_{k}^{1:M}|y_{1:k}\right)=p\left(c_{1:k}|X_{k}^{1:M},y_{1:k}\right)\overset{M}{\underset{m=1}{\prod }}p\left(X_{k}^{m}|y_{1:k}\right).$$ (13)

We can approximate the right-hand side of Eq. 13 using a Rao-Blackwellized particle filter. In this filter, each particle maintains its own joint target location distribution described in Eq. 12, given a certain history of data associations. Collectively, the particles approximate the distribution over the history of correspondences.

Typically, the state is factored such that an optimal, closed form filter is used to reduce the dimensionality of the problem (Doucet et al. (2000)). Common choices for the closed form filter are the Kalman filter or the Hidden Markov Model (HMM) (Ahmed et al. (2017)). We found that we had sufficient computational power for each particle to maintain a bank of particle filter trackers as described in Section 2 and therefore chose not to discretize the problem to fit an HMM. The computational cost of this formulation is *O*(*HMN*), where *M* and *N* are as defined above, and *H* is the number of history particles. Our approach has an additional benefit that with a continuous state space, the road network of interest could be expanded without increasing the number of discrete states or reducing the discretization resolution, as would have been necessary with an HMM. Additionally, we are not bound to a linear Gaussian model, as with a Kalman filter.

### 3.3 Data Association Sampling

Assuming that targets are otherwise indistinguishable, data association must be determined from the estimated state of the targets. One approach is maximum likelihood (ML) association, where the best fit is chosen as

$${\hat{c}}_{k}=\underset{m}{arg\;max}\;p\left(y_{k}|c_{k}=m,{\hat{c}}_{1:k-1},X_{k}^{m},y_{1:k}\right).$$ (14)

We instead use Data Association Sampling (DAS) (Thrun et al. (2006)), where data associations are sampled from a categorical distribution according to their likelihoods as

$$p_{c_{k}=m}\propto p\left(y_{k}|c_{k}=m,{\hat{c}}_{1:k-1},X_{k}^{m},y_{1:k}\right).$$ (15)

This can be approximated by summing the measurement likelihood over all particles for a given target and nomalizing to get

$$p_{c_{k}=m}\approx \eta \overset{N}{\underset{n=1}{\sum }}p\left(y_{k}|x_{k}^{m,n}\right),$$ (16)

Where Eq. 10 is used as the summand and $x_{k}^{m,n}$ is the *n*th particle for the *m*th target at time *k*. This approach can better retain multiple data association histories that have similar likelihood until they can be discriminated using later measurements.

The RBPF allows the UAV to properly associate measurements to targets, even when they leave and re-enter the camera field-of-view. However, these estimation techniques alone are not sufficient to maintain a good estimate of where all the targets are at any given time. The information from the filter must be used to feed a path planning algorithm so that the UAV can position itself to maximize target location certainty. The next section describes our approach to tracking and following multiple targets.

## 4 Single UAV Predictive Path Planning

When tracking multiple targets, the UAV should not simply find and follow one of them, but should spend time monitoring each target. We propose a path planning algorithm to maximize the information gain over all targets as the UAV flies above the road network. In Subsection 4.1 we show how djikstra’s algorithm is used to determine the overall cost of a given path based on the length of the path and how many particles are predicted to be encountered. We use the reward function from Subsection 4.1 to define an exhaustive receding horizon controller in Subsection 4.2. A neural net trained with Deep-RL is discussed in Subsection 4.3 as an alternative path planner.

### 4.1 Dijkstra’s Algorithm

Dijkstra’s algorithm finds the shortest path between two locations in a graph (Skiena (2008)). We apply Dijkstra’s algorithm by building a spanning tree of the road network, from which the UAV identifies the shortest path to any desired location. This paper modifies Djikstra’s algorithm to find a path of a desired length that will maximize the information gained during flight. A naïve approach for maximizing information takes the number of particles on an edge and divides by the length of the edge ‖G(e)‖

$$V_{k}^{e}=\frac{1}{\left\|G\left(e\right)\right\|}\overset{M}{\underset{m=1}{\sum }}\overset{N}{\underset{n=1}{\sum }}\delta \left(e^{m,n},e\right),$$ (17)

Where

$$\delta \left(e^{m,n},e\right)=\left\{\begin{array}{cc} 1,\; & \text{if }e^{m,n}=e\\ 0,\; & \text{otherwise}. \end{array}\right.$$ (18)

The UAV then feeds the edge values $V_{k}^{e}$ into a receeding horizon controller to find the path that maximizes target surveillance.

**FIGURE 2:**
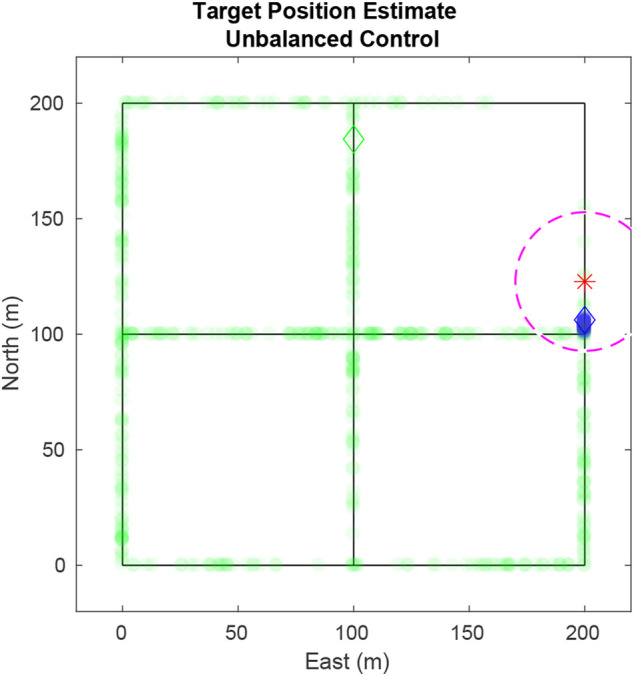
Using the naïve value function in Eq. 17, the UAV exclusively tracks a single target because of the closely packed particles of that target. Ideally the UAV should weight the target with highest entropy more heavily. Particles are plotted as transparent dots to indicate density. The diamonds shows true target positions, the star shows the UAV position, and the dashed circle delineates the UAV’s field-of-view F One target is represented in blue while the other is green.

Using this method presents a severe vulnerability because targets with low entropy have closely spaced particles. In the event of a large disparity between the entropy of targets the UAV will prioritize following the target with the lowest entropy to the detriment of all other targets. This situation is illustrated in Figure 2 where all the particles for the blue target are located on one edge giving it a higher priority over the green target whose particles are distributed evenly across the road network.

We avoid the above scenario by implementing target weighting based on the entropy *H* ^*m*^ of a target estimate. Entropy-based weighting allows the UAV to prioritize tracking high entropy targets without having the entropy of other targets grow unchecked. The entropy (Shannon (1948)) of particles $X_{k}^{m}$ is given by

$$H_{k}^{m}=\overset{N}{\underset{n=1}{\sum }}p\left(x_{k}^{m,n}|y_{1:k}\right)\log p\left(x_{k}^{m,n}|y_{1:k}\right).$$ (19)

To get the target weighting, first normalize the entropy *via* *η*_*u*,*k*_ and then apply the sigmoid function

$$\gamma_{k}^{m}=\frac{1}{1+e^{-a\left(\eta_{u,k}H_{k}^{m}-0.5\right)}},$$ (20)

Where *a* is a gain defining how strongly target weights get pushed apart by small differences in entropy. Targets with higher entropy are given higher weights. The resulting weighted edge value is

$$V_{w,k}^{e}=\frac{1}{\left\|G\left(e\right)\right\|}\overset{M}{\underset{m=1}{\sum }}\gamma_{k}^{m}\overset{N}{\underset{n=1}{\sum }}\delta \left(e^{m,n},e\right),$$ (21)

**FIGURE 3:**
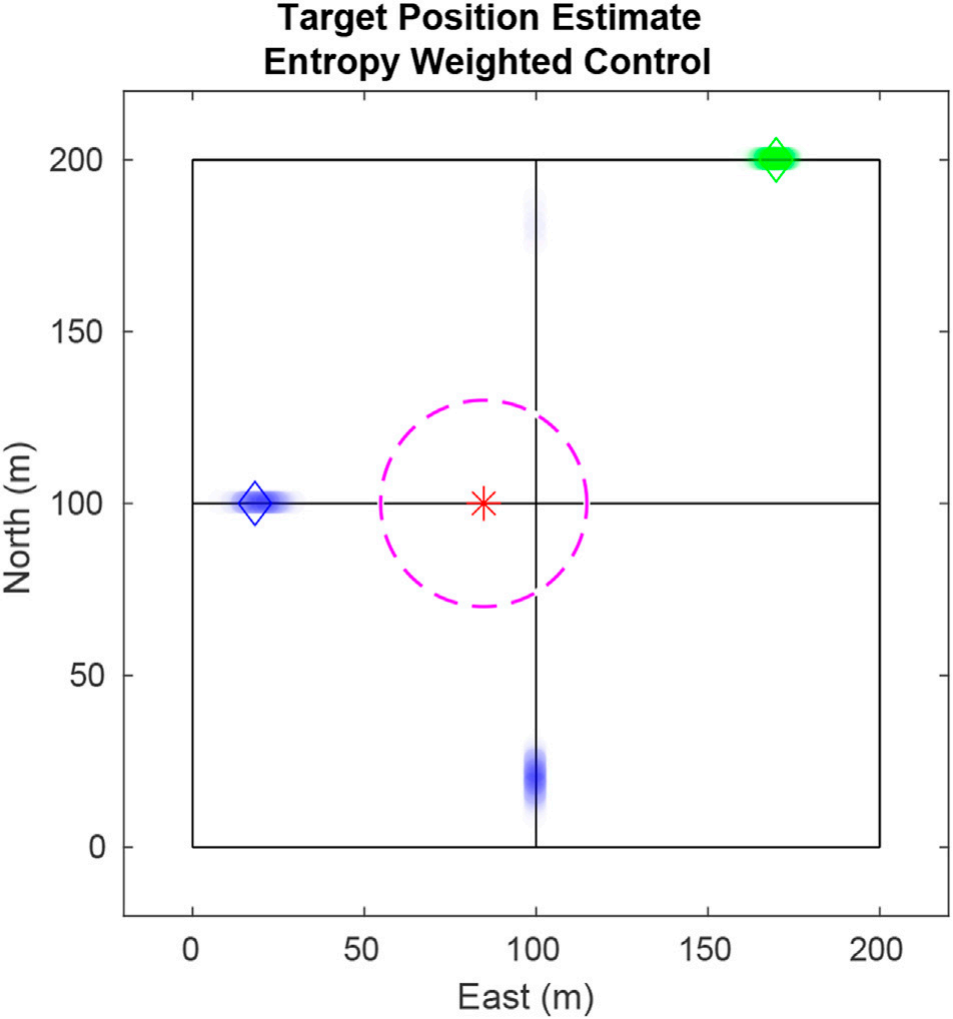
Using edge values weighted by target entropy, the UAV prioritizes the blue particles over the green since the entropy of blue is larger than that of green.

Resolving the issue shown in Figure 2 that used Eq. 17. In Figure 3, using Eq. 21, the UAV pursues the blue target as it has a greater weight than the green target. Without entropy weighting, the UAV would return to track the green target, allowing knowledge of the blue target to dissipate. With entropy weighting the UAV attempts to maintain equal certainty across all targets.

We next extend this strategy to receding horizon control by propagating the particles forward until the time the UAV will be traversing each edge.

### 4.2 Exhaustive Receeding Horizon Control

The UAV needs to account for particle movement while path planning in order to achieve an optimal path. The receding horizon controller creates a tree structure where each branch represents a potential path for the UAV. Branch weights are determined by the weighted edge values $V_{w,k}^{e}$ along that branch path. Particle propagation must be taken into account since the edge value is a function of time and the UAV does not travel instantaneously down the path. The tree is built recursively where a breadth first search is performed down the tree structure while accounting for particle movement. Each point of exploration of the breadth first search is referred to as a lookahead step.

In each lookahead step, a branch is created for each edge leaving the current node. The edge value, Eq. 21, is added to that branch, and the particles on that edge are removed from further consideration along that path. All remaining particles are propagated forward by the amount of time it takes the UAV to traverse that edge. This process repeats for each lookahead step until the maximum number of lookaheads are performed. The branch with the highest value is found, and the UAV traverses the first edge of that path before repeating the procedure to calculate the next edge to traverse.

The computational cost of this algorithm is *O*(*MNd* ^*L*^), where *M* is the number of targets, *N* is the number of particles per target, *d* is maximum number of edges leaving a node, and *L* is the number of lookahead steps. This path planning technique was shown in simulation to be effective in choosing good paths.

### 4.3 Deep-RL

We also implemented a path planner that uses a neural net trained using deep reinforcement learning. The neural net offers an on-line computational advantage over other path planners since the execution of the neural net is *O*(1) after training. While this is an ideal computational cost, the trade off comes because the neural net must be trained which, depending on design, map size and number of targets, can take an unacceptably long time. In addition, tuning the training parameters can be a non-trivial process.

In this paper the neural net is trained using a proximal policy optimization (PPO) (Schulman et al. (2017)). PPO works by taking the loss gradient and uses first order optimizers to maintain a gradient descent thereby optimizing the objective of the net. The top layer of the neural net matches the size of the feature space with each subsequent layer taking roughly two thirds the size until the bottom layer, which is the size of the action space. In order to train the neural net, we translate the state of the map, UAV positions, and particle data into a numerical layout that is readable by the net. This is the feature space.

The feature space has five distinct sets of information. First in the state space are the positions of the *Q* UAVs, $A_{x,y}^{q}$ in *x*, *y* coordinates where *q* ∈ [1, *Q*]. Combined with the map layout, the neural net is able to learn which actions in the action space are valid for each UAV’s location.

The particle distribution is the next part of the state space, given by

$$X_{1:B}^{m}=\left[X_{1}^{m},\ldots ,X_{B}^{m}\right].$$ (22)

The map is separated into *B* equal sized bins of 10 m. The number of particles from target *m* in *b* = 1 is $X_{1}^{m}$ . In this way the path planner knows which actions would be best to minimize the entropy for each individual target.

To know which target’s entropy we need to minimize we then append a list of each target’s entropy weighting $\gamma_{k}^{m}$ , i.e.

$$\gamma_{k}^{1:M}=\left[\gamma_{k}^{1},\ldots ,\gamma_{k}^{M}\right],$$ (23)

allowing the neural net to focus on targets with greater entropy.

Finally, the map layout is included as the list of nodes

$$N_{x,y}^{1:C}=\left[N_{x}^{1},N_{y}^{1},\ldots ,N_{x}^{C},N_{y}^{C}\right].$$ (24)

This provides the map’s *x*, *y* coordinates when combined with their connecting edges,

$$E^{1:f}=\left[E^{1},\ldots ,E^{f}\right].$$ (25)

Combining these five sets of information, the feature space is defined as the one dimensional vector,

$$F_{\text{net}}=\left[A_{x,y}^{1}\ldots A_{x,y}^{Q},X_{1:B}^{1}\ldots X_{1:B}^{M},\gamma_{k}^{1:M},N_{x,y}^{1:C},E^{1:f}\right].$$ (26)

**FIGURE 4:**
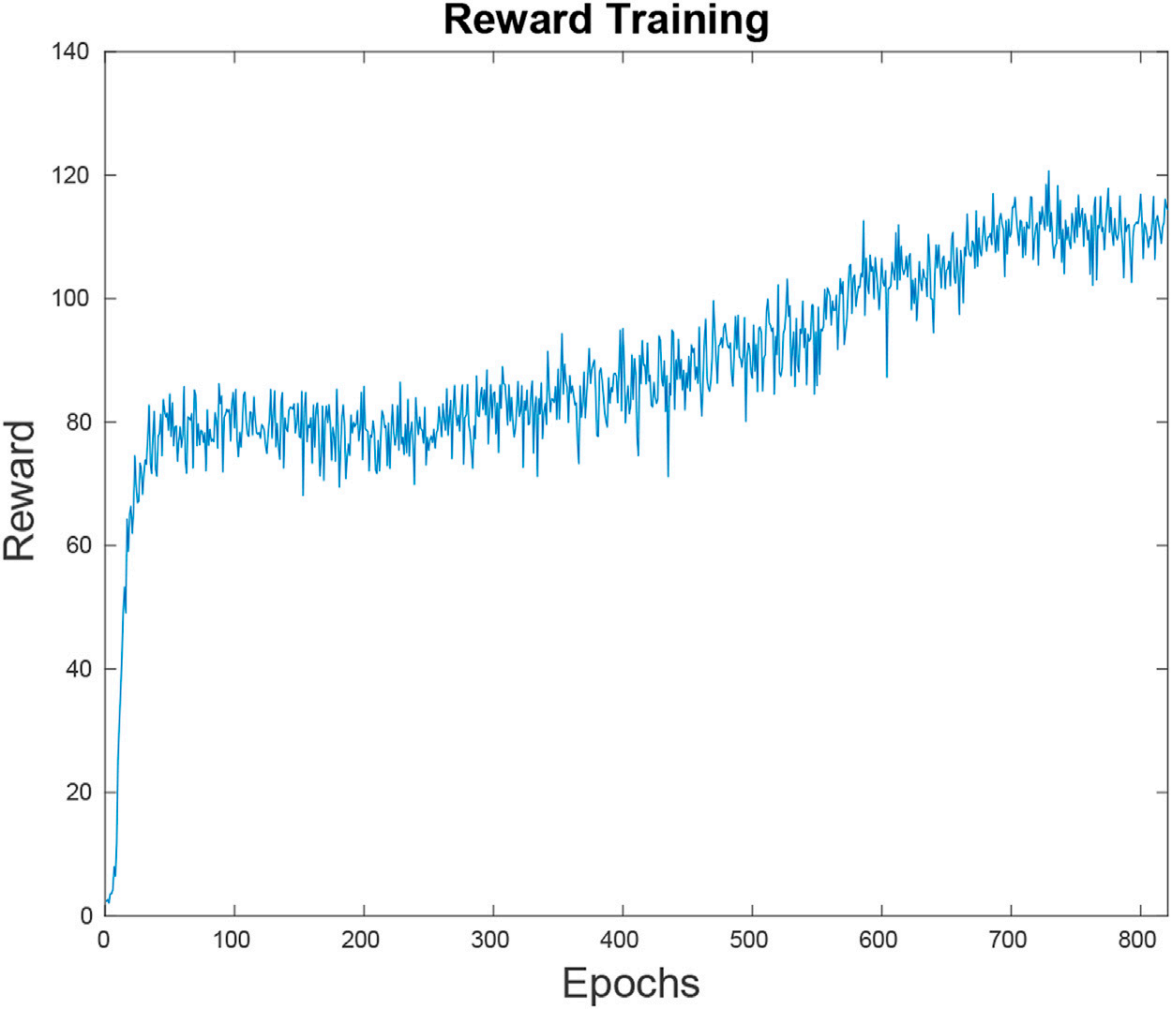
This figure depicts the neural net’s reward function, or the inverted loss function over the training time. Within the first 80 epochs the neural net has reached an average reward of 80 U on random simulations. At this point it is outperforming a random path planner but the results are not competitive. Since the learning has hit a plateau, it takes 500 more epochs to bridge the gap to the point where it is performing at the level shown in Figure 11.

Using an entropy-based reward function $R=\exp\left(\gamma_{\text{max}}-\gamma_{k}\right),$ we trained the net to reasonable performance levels within a short span. The training cycles after the initial rise only yielded incrementally better results, as seen in Figure 4. There are many different variables that go into the speed and accuracy with which a neural net can be trained. Future work would involve optimizing the neural net’s parameters to prevent it from plateauing after 80 epochs. Ideally, it would maintain its steep learning curve until it maximizes the reward function.

Using this method the neural net is trained to work on a specific map with a set number of targets. However, with recent advances done in transfer learning (Banerjee and Stone (2007)) it may be possible to train a neural net capable of working in various scenarios and road network configurations. Transfer learning uses the knowledge gained in solving one problem when learning how to solve another problem. Rather than training on all possible maps simultaneously we would train the neural net on a single map and use the principles of transfer learning to augment the current neural net to work for multiple maps.

## 5 Lower Bound for Average Entropy of RBPF

In this section we develop theoretical results and a computable algorithm to find the lower bound of the average entropy of the Rao-Blackwellized Particle Filter (RBPF). Throughout this section we use *Q* to denote the number of UAVs, *δ*_*t*_ to denote the time step duration, and *T* to denote the average time it takes a single UAV to take a round trip in the road network, i.e., the average time it takes a UAV to return to its current location, while traversing the network.

Our results depend on a number of assumptions that we outline below.

Assumption 1. *The velocities of the targets and the UAVs at each time step are drawn from a Gaussian distribution with constant mean.*

Assumption 2. *The round-trip path is the ideal layout for the multi-agent path planner.*

Assumption 3. *Each target vehicle uses an independent stochastic path planner.*

Assumption 4. *Targets are equally likely to be at any point on the road network.*

In the scenarios considered, the targets have a uniform likelihood of starting at any location in the map. They maintain a constant velocity and use independent stochastic path planners which result in a uniform likelihood of the targets being at any location on the road network.

Assumption 5. *The average distance between any two graph nodes* *S* *is equal to the average distance between any two points on the graph multiplied by a scale factor* *αS* *.*

The distance between any two points on the graph can be found in one of two ways. If the points are on the same edge then the distance is the Euclidean distance between the points. Otherwise the distance is the minimum distance between the nodes adjacent to the two points plus the distance from the points to the adjacent nodes. Because targets are equally likely to be at any location on the map, averaging these two scenarios results in a distance that is closely related to the average distance between nodes. However as the exact relationship between the average distance between points on a graph and the average distance between modes is dependent on individual map configuration it must be determined on a map by map basis.

We verified Assumption 5 using Monte Carlo simulations. With 1000 Monte Carlo runs per map over 144 maps that varied from 100 to 3600*m* ^2^ in size and found that the error between the Dijkstra’s average and the distance between two points was less than two percent of the given map size showing that for a wide variety of maps the scale factor *α* between *S* and any two points on the graph is relatively close to one.

Assumption 6. *Particle filter resampling occurs at each timestep. The particle filter receives one positive update per sighting and no negative updates.*

Negative updates affect the entropy of the filter any time the UAV is in the proximity of particles, but the target is not yet in sight. While we can determine what the average round-trip time is, the round-trip path varies making it difficult to account for how negative updates will affect the particle filter on average.

Assumption 7. *Mode merging does not occur.*

A mode is comprised of particles that are in close proximity and have similar velocities. A detailed description of the evolution of modes is provided in Subsection 5.3.

We now provide a theorem that defines the lower bound on the average entropy. This theorem uses the degree type *D* of a mode in the entropy calculation. The degree type is the *D* percent of the particle filter contained in that mode. For example, when a mode of degree *D* = 100*%* exists, every particle in the filter is in that mode.

Theorem 1. *Suppose that* *Q* *is the number of UAVs, that* *T* *is the average time it takes a UAV to traverse a loop in the map, and that* *δ*_*t*_ *is the time step duration*

*The lower bound for average entropy of a RBPF governed by Assumptions* 1–7 *is*

$$B_{L}=\frac{1}{T_{Q}}\overset{T_{Q}}{\underset{k=0}{\sum }}E\left[H_{k}\right]$$ (27)

Where

$$T_{Q}=\frac{T}{Q\delta_{t}}$$ (28)

*Is the average minimum number of timesteps to visit a target (average minimum round-trip time* *T* *divided by the number of UAVs* *Q* *),* *δ*_*t*_ *is the timestep duration, and*

$$E\left[H_{k}\right]=-\underset{D}{\sum }\overset{H}{\underset{h=0}{\sum }}P_{d,k}\varphi_{k}\left[b^{h}\right]\log\left(D\varphi_{k}\left[b^{h}\right]\right)$$ (29)

*Is the expected entropy, where* *φ*_*k*_[*b* ^*h*^] *is the probability of a target existing in a discrete section* *b* ^*h*^ *of the map,* *D* *is the degree type, and* *P*_*D*_ *is the probability of a mode of degree type* *D* *existing.*

The proof depends on five lemmas which are presented in the subsequent subsections. Lemma 1 identifies the average minimum round-trip time *T* to visit all targets on the map. The variance growth of the RBPF is defined in Lemma 2 and the average variance after a positive update is defined in Lemma 3. The relationship between variance and entropy in a single mode is in Lemma 4. And the probability *P*_*D*_ of a mode of degree type *D* existing is given in Lemma 5.

### 5.1 Minimum Time to All Targets

In this section we provide a formula for calculating the average minimum-time to visit all the targets on the road network. Let G=(N,E) be a graph representing the road network, and let *S*_*ij*_ be the minimum distance path between nodes $n_{i}\in N$ and $n_{j}\in N$ , and let len(*S*_*ij*_) be the length of *S*_*ij*_. Let *D* be the diameter of the graph, and let $S_{i}$ denote the set of minimum paths that contain exactly *i* edges, and let $S=\cup_{i=1}^{D}S_{i}$ be the set of all minimum length paths between nodes. We have the following lemma.

Lemma 1. *Let* *M* *be the number of targets, and assume that Assumptions* 1*,* 4*, and* 5 *hold. Then the average distance between targets on a round-trip path is given by*

$$S_{\text{avg}}\left(M\right)=S_{\text{point}}\left(\frac{M}{M-1}\right)\overset{M-1}{\underset{m=1}{\sum }}\frac{\sin\left(\frac{\pi }{M}\right)}{\sin\left(\frac{\pi m}{M}\right)},$$ (30)

*Where* *S* *_point_* *is the average distance between any two points on the graph, given by Assumption* 5 *as the average distance between nodes, and the average minimum round-trip time to visit all targets is*

$$T_{\text{ave}}\left(M\right)=\frac{S_{\text{avg}}\left(M\right)}{V_{a}},$$ (31)

*Where* *V*_*a*_ *is the airspeed of the UAV.*

Proof.

**FIGURE 5:**
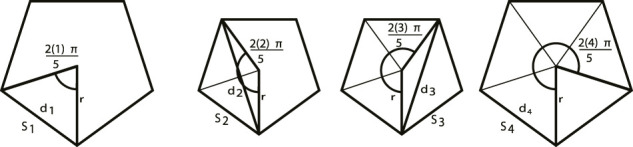
In the case of five equally-spaced targets the shortest round-trip distance is a regular polygon. These four polygons represent all possible polygon sizes. Taking the side length *S*_*avg*_ gives us the average distance between targets on the minimum round-trip path shown in Eq. 30.

By Assumption 4 and 5 each target is on average *S*_point_ meters apart when travelling along the road network. The minimal path that visits *M* targets that are *S*_point_ meters apart is given by a regular polygon with a target located at each vertex. The distance between vertices is equally likely to be *S*_point_. Since the diagonals and the edges of a regular polygon are not equidistant this results in a set of equally likely polygons of varying sizes, as shown in Figure 5. The perimeters of this set of polygons are averaged together to estimate the average round-trip distance.

For an isosceles triangle with equal sides of length *r*, base of length *D* and apex angle *θ*, we have *D* = 2*r* sin(*θ*/2). Therefore perimeter length of a regular polygon with *M* sides and radius *r* is

$$S\left(r,M\right)=2Mr\sin\left(\frac{\pi }{M}\right).$$

When nodes of the polygon are separated by *m* sides, the angle separating the nodes is 2*πm*/*M*. When the distance between those nodes is *S*_point_, then the radius is

$$r_{m}=\frac{S_{\text{point}}}{2\sin\left(\frac{\pi m}{M}\right)},$$

And the perimeter of the associated polygon is

$$S\left(r_{m},M\right)=MS_{\text{point}}\frac{\sin\left(\frac{\pi }{M}\right)}{\sin\left(\frac{\pi m}{M}\right)}.$$

Averaging over all polygons *m* = 1, *…*, *M* − 1 gives Eq. 30. The average time duration of a round trip across all targets is then given by the average distance divided by the UAV’s airspeed as shown in Eq. 31. QED.

Figure 5 illustrates the set of possible polygons when there are five targets. Since any two targets are an average distance of *S* apart, the lines between two vertices (*d*_1_, *d*_2_, *d*_3_, and *d*_4_) remain a set distance, but the side lengths *S*_*m*_ for *m* = 1, *…*, 4 change.

We note that all quantities in this section are readily computable. Given a road network, Dijkstra’s algorithm can be used to find the minimum length path between every two nodes. These can be averaged to find *S*_node_. The number of edges and the average length of each edge are easily computed, and therefore *S*_point_ can be computed. *S*_ave_(*M*) therefore follows from knowing the number of targets *M*.

### 5.2 Single Mode Entropy

In this subsection we provide a formula for calculating the particle filter’s entropy when there is only one mode. The entropy of a mode at time *k* can be calculated from its variance. This is because entropy in the particle filter is based off the particle filter density, which is directly related to the variance when there is only one mode. Because the measurement model uses Gaussian noise on the velocity, the modes in the particle filter are also Gaussian. Entropy is determined from the particle’s density, represented by the positional variance $\sigma_{p,k}^{2}$ . The mode’s position in the graph is irrelevant.

Until a mode encounters an intersection causing it to split into multiple modes its behaviour and variance are the same as would occur in a Kalman Filter. To predict $\sigma_{p}^{2}$ at time *k* we need to know how the mode’s variance grows over time and what the variance will be after a positive measurement update. In this section, the variance right after incorporating a positive measurement update will be referred to as the minimum variance.

We give the calculation for the single mode entropy by first describing the variance growth of the RBPF between updates (Lemma 2), then computing the average variance after a positive update (Lemma 3), and finally providing the relationship between variance and entropy in a single mode (Lemma 4).

Lemma 2. *Given Assumptions* 1 *and* 6*, the growth of the positional variance between sightings for a single mode is*

$$\sigma_{p,k}^{2}=\sigma_{p,k_{0}}^{2}+\sigma_{v}^{2}\delta_{t}^{2}\left(k-k_{0}\right),$$ (32)

*Where* *k*_0_ *is the time of the last sighting and* *δ*_*t*_ *is the timestep, and* *k* ≥ *k*_0_ + 1*.*

Proof.

The behaviour of a single mode between updates matches that of a Kalman filter. Therefore, we use the Kalman filter’s time propagation equation to predict a mode’s variance growth. At time *k*_0_ the Kalman filter’s co-variance matrix is

$$P_{k_{0}}=\left(\begin{array}{cc} \sigma_{p,k_{0}}^{2} & 0\\ 0 & \sigma_{v}^{2} \end{array}\right).$$ (33)

Under Assumption 1, the state transition matrix for a constant-velocity target is

$$F=\left(\begin{array}{cc} 1 & \delta_{t}\\ 0 & 1 \end{array}\right).$$ (34)

Propagating the covariance using *P*_*ℓ*+1_ = *FP*_*ℓ*_ *F* ^*⊤*^ for one time step gives

$$P_{k_{0}+1}^{\text{pre}-\text{resample}}=\left(\begin{array}{cc} \sigma_{p,k_{0}}^{2}+\delta_{t}^{2}\sigma_{v}^{2} & \delta_{t}\sigma_{v}^{2}\\ \delta_{t}\sigma_{v}^{2} & \sigma_{v}^{2} \end{array}\right).$$ (35)

However, resampling according to Assumption 6 removes the cross-covariance between position and velocity resulting in

$$P_{k_{0}+1}=\left(\begin{array}{cc} \sigma_{p,k_{0}}^{2}+\delta_{t}^{2}\sigma_{v}^{2} & 0\\ 0 & \sigma_{v}^{2} \end{array}\right),$$ (36)

Implying Eq. 32 after *k* − *k*_0_ samples. QED.

In the next lemma we incorporate measurement updates to calculate the average minimum variance for a mode. The average duration between measurement updates is the average minimum round-trip time computed in Eq. 31.

Because the particles are constrained to the road network they are effectively constrained to 1-D space. The UAV measures the target’s position along the road segment, making the measurement covariance *R* a scalar.

Lemma 3. *Given Assumptions* 1*,* 4*, and* 6*, the average minimum variance* $\sigma_{p_{0}}^{2}$ *for a single mode is*

$$\sigma_{p_{0}}^{2}=\left(\frac{T_{\text{ave}}\left(M\right)\delta_{t}\sigma_{v}^{2}}{2}\right)\left[\sqrt{1+\frac{4R}{T_{\text{ave}}\left(M\right)\delta_{t}\sigma_{v}^{2}}}-1\right].$$ (37)

Proof.

The minimum variance occurs after a positive update and is a function of both the variance of the mode prior to the update and the measurement covariance. The relationship between the variance before and after an update can be expressed in the information update equation from the information filter as (Brown and Hwang (1997))

$${\left(P_{k}^{+}\right)}^{-1}={\left(P_{k}^{-}\right)}^{-1}+H^{⊤}R^{-1}H,$$ (38)

Where *H* = (1, 0) is the observation model. From Eq. 32, the (1, 1)-element of Eq. 38 in steady state is given by

$$\frac{1}{\sigma_{p_{0}}^{2}}=\frac{1}{\sigma_{p_{0}}^{2}+T_{\text{ave}}\left(M\right)\delta_{t}\sigma_{v}^{2}}+\frac{1}{R}.$$

Rearranging, we get the quadratic form

$$\sigma_{p_{0}}^{4}+\sigma_{p_{0}}^{2}T_{\text{ave}}\left(M\right)\delta_{t}\sigma_{v}^{2}-RT_{\text{ave}}\left(M\right)\delta_{t}\sigma_{v}^{2}=0,$$

Where the positive solution is

$$\sigma_{p_{0}}^{2}=\frac{-T_{\text{ave}}\delta_{t}\sigma_{v}^{2}+\sqrt{{\left(T_{\text{ave}}\delta_{t}\sigma_{v}^{2}\right)}^{2}+4RT_{\text{ave}}\delta_{t}\sigma_{v}^{2}}}{2},$$

From which we get Eq. 37. QED.

Lemma 4. *For the* *kth* *mode in the particle filter, the entropy is lower bounded by*

$$H_{k}\geq \log_{2}\sqrt{2\pi e\sigma_{p_{0}}^{2}},$$ (39)

*Where* $\sigma_{p_{0}}^{2}$ *is given in* *Eq.* (37) *.*

Proof.

By straightforward calculus, the entropy of a Gaussian distribution $f\left(x\right)=1/\sqrt{2\pi \sigma^{2}}\exp\left(-\frac{{\left(x-\mu \right)}^{2}}{2\sigma^{2}}\right)$ is computed as

$$H\left(f\left(x\right)\right)=-\int_{x=-\infty }^{\infty }f\left(x\right)\log_{2}f\left(x\right)\;dx=\log_{2}\sqrt{2\pi e\sigma^{2}},$$

Where the entropy is taken over an infinite interval. For a finite road network, the tails of the distribution are folded back into the network thereby increasing the uncertainty over the network implying that entropy increases. Therefore, Eq. 39 is the lower bound on the entropy for each mode, which is given by a Gaussian distribution with variance in Eq. 37. QED.

From a practical standpoint, the entropy of the particle filter is computed as

$$H_{k}\approx \overset{H}{\underset{h=0}{\sum }}\varphi_{k}\left[b^{h}\right]\log\varphi_{k}\left[b^{h}\right],$$ (40)

Where the map is discretized into H bins of 1 m length $b^{0:H}=\left[0,1,2,\ldots ,L_{map}\right],$ , where *L*_*map*_ is the length of the map, and *φ*_*k*_[*b* ^*h*^] is the probability of a target existing in section *b* ^*h*^ of the map.

### 5.3 Estimated Number of Modes

**FIGURE 6:**
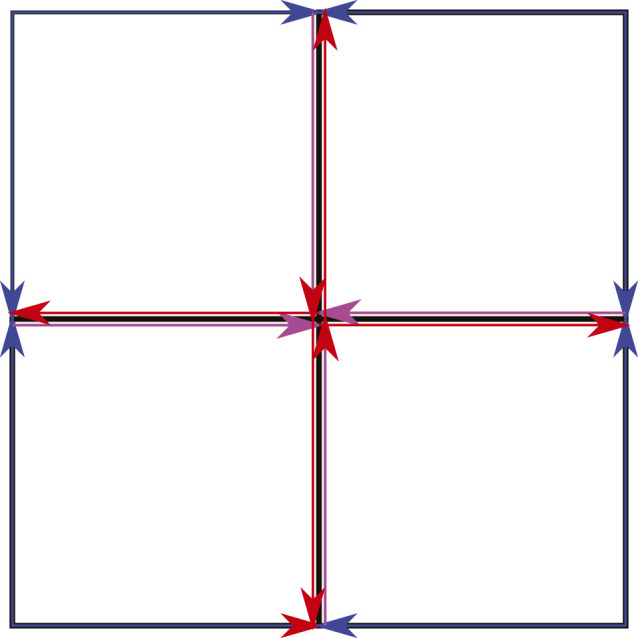
Map Groupings.

**FIGURE 7:**
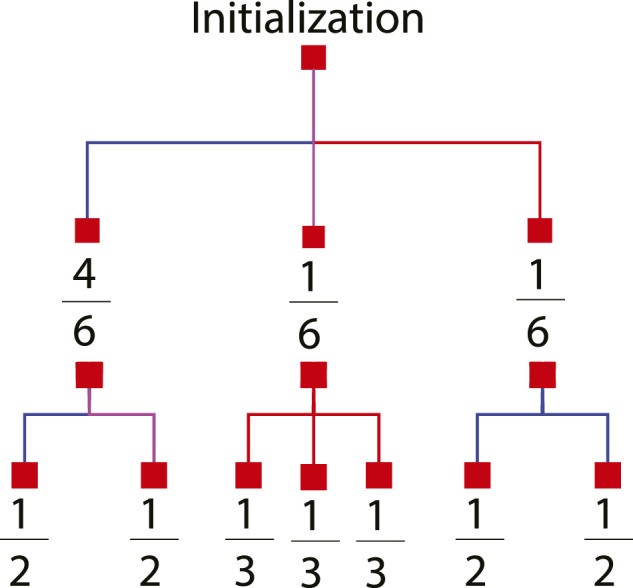
Branching probabilities. Mode Splitting Analysis for a 3 × 3 map. A 3 × 3 map is a grid comprising of nine nodes in a 3 × 3 layout with each edge equidistant.

To estimate a mode’s probability of existence *P*_*D*_ for each degree type *D* over time, the map is analyzed to understand how long a single mode will traverse each map’s edge and how many modes it will split into once it leaves the edge. Each edge may be considered separately to understand the modes behavior. However, we will exploit the similarities found in many maps by grouping edges (or contiguous edges that don’t split into multiple road segments) if they have the same length and relate to the rest of the map in an identical fashion. These groups are termed subdivisions. In the case of the 3 × 3 map, shown in Figure 3 the map is composed of sixteen directed edges. The edges can be grouped into three subdivisions as shown in Figure 6 by the blue, red, and purple colored arrows. A map’s subdivisions are then converted into the tree structure shown in Figure 7. The first set of branches show the probability that a target will be initialized in each subdivision while the second set of branches show which subdivisions are accessible from the current subdivision, how many modes will be spawned upon leaving the subdivision, and the percent of the current mode that will exist in each subsequent mode. In the case of a stochastic path planner, the parent mode will divide into equal portions when splitting into children modes.

When a mode is initialized it has a degree type *D* = 100*%*. The way it splits into children modes is dependent on the subdivision it was initialized in. Each subsequent mode’s *P*_*D*_ is dependent on the *P*_*D*_ of its immediate parent and the time it takes for the parent to traverse its subdivision. Though complex, mode probabilities deterministically depend on the properties of the subdivision in which it was initialized.

Lemma 5. *Under Assumptions* 1*,* 3*, and* 7*, the probability* *P*_*D*_ *of each mode of degree type* *D* *existing at time* *k* *is given by Algorithm* 2 *listed below.*

**Algorithm 1** GetModeSplitLevel(*sd*, *P*_init_, *T*_*r*_, *D*, *T*_*s*_, Map, *k*)

**Input:** the current subdivision *sd*, Probability of first parent

$P_{\text{init}}\in R$ , the time it takes to traverse the first parent’s subdivision
*T*_*r*_, the current mode degree *D*, the time the current mode came into existence *T*_*s*_, the Map in tree format (shown by Figure 7), and the current time *k*.
**Output:** The mode probability for each of the children of the current mode
*P*_*D*_ and the list of child modes organized by degree *D* added to the unprocessed modes list *U*.
1: *U* ←{}
2: **for** option ∈ options **do**
3: *D*_next_ ← *D*/len(Map[option].neighbors)
4: *T*_*o*_ ← Map[*sd*].offsets[option]
5: *T*_*d*_ ← Map[option].offsets
6: *P*_*D*_[*D*_next_] ← *P*_init_ *DW*(*k*, *T*_*r*_, *T*_*s*_ + *T*_*o*_, *T*_*d*_)
7: unprocessed[*D*_next_] append

$\left[P_{\text{init}},T_{r},T_{s}+T_{o},\right.$ option
8: **end for**
9: **return** *P*_*D*_, *U*

**Algorithm 2** GetModeProbabilities(*D*_*c*_, *U*_*c*_, *k*, Map, *T*_*Q*_)

**Input:** the list of initial mode degrees *D*_*c*_, the list of initial modes
*U*_*c*_, the current time *k*, the map in tree format shown in Figure 7, the maximum number of timesteps *T*_*Q*_.
**Output:** the probability for each mode degree type *P*_*D*_.
1: **for** *D* ∈ *D*_*c*_ **do**
2: **for** line ∈ *U*_*c*_ **do**
3: *sd* ← line.sd
4: *P*_*init*_ ← line.prob
5: *T*_*r*_ ← line.range
6: *T*_*s*_ ← line.start
7: **if** *T*_*s*_ < *T*_*Q*_ **then**
8: ModeProb,*U*_*c*_ ← GetModeSplitLevel(*sd*,
*P*_*init*_, *T*_*r*_, *D*, *T*_*s*_,
Map, *k*)
9: **end if**
10: **end for**
11: **end for**
12: **if** *U*_*c*_ ≠ ∅ **then**
13: *D*_*n*_ ← *U*_*c*_.keys
14: ModeProb ←
GetModeProbabilities(*D*_*n*_, *U*_*c*_, *k*,Map,*T*_*Q*_)
15: **endif**
16: **return** ModeProb

Proof.

When a mode is initialized in a given subdivision at time *k*_0_ it has a uniform likelihood of being anywhere in that subdivision. As a result the earliest that mode can leave the subdivision is at *k*_0_ + 1 (assuming it was initialized at the end of the subdivision) and the latest is at *k*_0_ + *T*_*r*_, where *T*_*r*_ is the time it takes a target to traverse the subdivision (assuming it was initialized at the beginning of the subdivision). After initialization, the progress of a mode is deterministic. Therefore the linear probability of a mode encountering its next intersection at time *k* is

$$w\left(k,T_{s},T_{r}\right)=\left\{\begin{array}{cc} 0\; & k<T_{s}\\ \frac{k-T_{s}}{T_{r}}\; & T_{s}\leq k<T_{s}+T_{r}\\ 1\; & otherwise \end{array}\right.,$$ (41)

Where *T*_*s*_ is the timestep that the mode came into existence and *T*_*r*_ is the time it takes to traverse the initial subdivision of the mode’s first parent.

Given Eq. 41 and Assumption 3 we can extrapolate the probability of existence for the children of a specific mode

$$W\left(k,T_{r},T_{s},\left[T_{d_{1}},\ldots ,T_{d_{F}}\right]\right)=w\left(k,T_{s},T_{r}\right)-\overset{F}{\underset{f=0}{\sum }}\frac{1}{F}w\left(k,T_{s}+T_{d_{f}},T_{r}\right)$$ (42)

Where *T*_*s*_ is the time the child modes come into existence and $\left[T_{d_{1}},\ldots ,T_{d_{F}}\right]$ are the times it takes each of the *F* child modes to traverse their respective subdivisions. The child modes’ probabilities are divided by *F* since, with Assumption 3, particles will traverse each edge with equal probability. Using these parameters, Eq. 42 first predicts the probability of the child modes coming into existence with *w*(*k*, *T*_*s*_, *T*_*r*_). Then it iterates over the *F* child modes to sum their probabilities of ceasing to exist. Combining these two halves of Eq. 42 gives the overall probability of existence at any time *k* for the child modes.

Algorithm 1 takes a single mode and calculates the probability of existence for each of its children *P*_child_ using Eq. 42. The *P*_*D*_ for the mode degree type *D* of the children is incremented in line 6 by combining *P*_child_ with the probability of the first parent *P*_init_ and normalizing by the mode’s degree. The algorithm then returns the *P*_*D*_ for the modes processed as well as a list of the child modes appended to the unprocessed modes list.

Algorithm 2 recursively calculates the probability *P*_*D*_ that each mode degree type *D* exists across a given duration. It calculates all unprocessed modes and inputs the ones that are born before time *T*_*Q*_ into Algorithm 1. The unprocessed modes returned from Algorithm 1 are appended to the unprocessed mode list. This recursion repeats until there are no remaining unprocessed modes within the desired time window. Algorithm 2 ensures each possible mode is passed into Algorithm 1 and Algorithm 1 calculates the mode’s *P*_*D*_. Therefore, Algorithm 2 provides the probability of *P*_*D*_ for each mode degree type *D* over a set duration *T*_*Q*_. QED.

We verified Lemma 5 by comparing it with results from 10,000 Monte Carlo runs on the map in Figure 6. For each Monte Carlo run a mode of degree tye *D* = 100*%* was initialized on a random location on the map and then allowed to traverse the map, propagating and splitting for a set time period *T*_*Q*_ = 20/*δ*_*t*_. The expected number of modes of degree type *D* obtained by averaging the mode count from the Monte Carlo runs was converted into the probability

$$P_{D}=\frac{E\left[D\right]}{D}.$$ (43)

**FIGURE 8:**
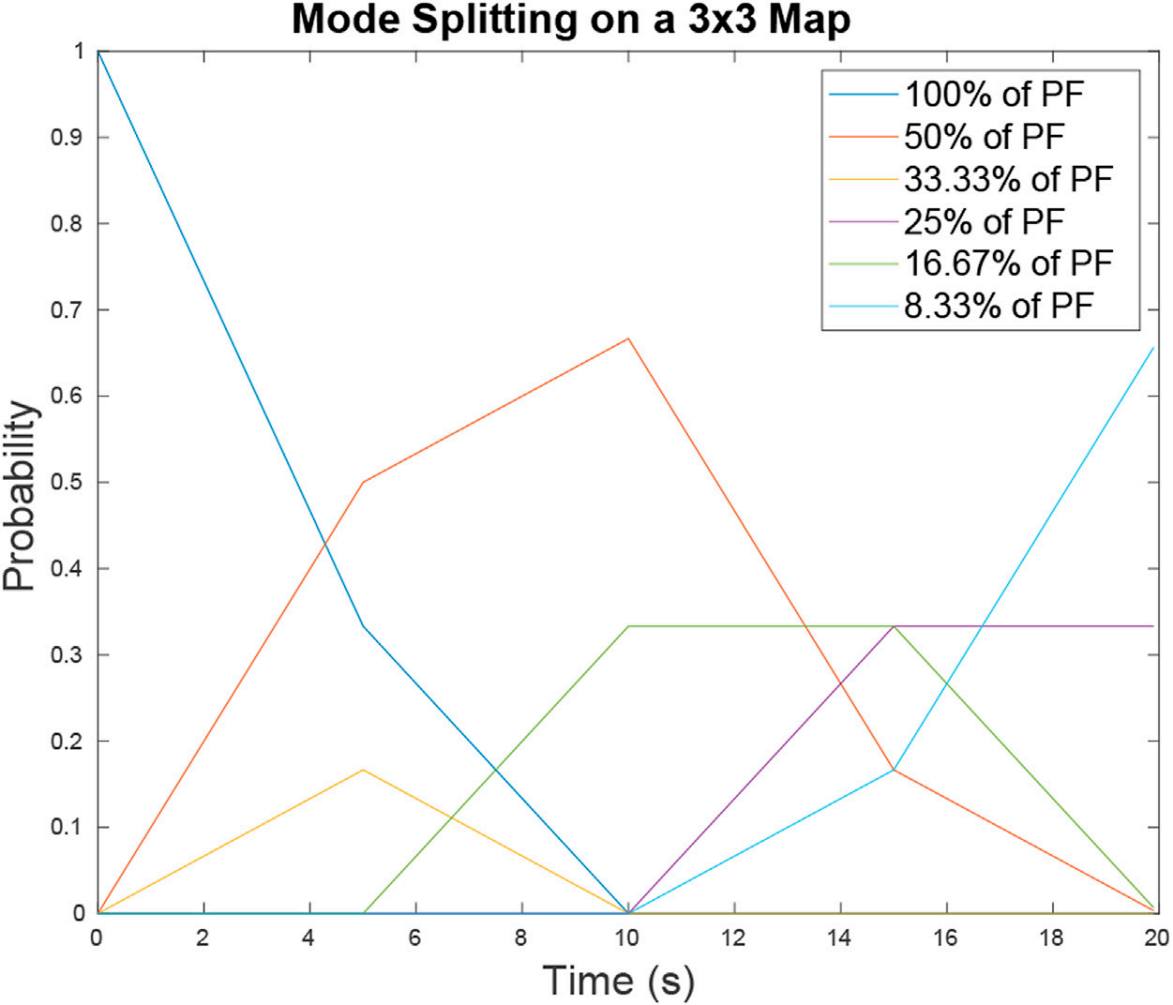
Probability of mode existence *P*_*D*_ for map 6 over time *t* = [0, 20] s. The probability of a mode that contains *D* percent of the particle filter is represented by the different lines plotted. As time progresses the probabilities of larger modes decrease as they evolve into smaller modes with increasing likelihood.

The resulting *P*_*D*_ matched the results of Algorithm 2. Plotting *P*_*D*_ for all possible degree types yields Figure 8. On large maps with many intersections there is a possibility that between target sightings the modes in the particle filter will merge as well as split over time. Mode merging is a complex subject as it can result in non-Gaussian structures depending on how the modes line up with each other as they merge. Mode merging is not analyzed in this paper but will be considered in future work.

### 5.4 Theorem for Lower Bound of Average Entropy

We are now prepared to prove Theorem 1.

**Proof of Theorem 1.** Lemma 1 gives the round-trip time to visit each target. Under Assumption 2 the ideal multi-agent path planner will evenly space the UAVs around the roundtrip path. The time between target sightings is obtained by taking the round-trip time given by Eq. 31 and dividing by the number of active UAVs resulting in Eq. 28.

The entropy of a single mode system is provided by Eq. 40, from Lemma 4, where *φ*_*k*_(*b* ^*h*^) assumes *D* = 100*%* to properly account for the all the particles belonging to that one mode. The mode degree then determines the percentage of *φ*_*k*_(*B* ^*h*^) that is used to calculate the entropy converting Eq. 40 into

$$u_{d,k}=\overset{H}{\underset{h=0}{\sum }}-d\varphi_{k}\left[b^{h}\right]\log\left(d\varphi_{k}\left[b^{h}\right]\right)$$ (44)

For a single mode of degree *d*.

The expected number of modes of degree type *D* is

$$E\left[D\right]=P_{D}\frac{1}{D}.$$ (45)

Which is the probability that that modes of type *D* exists divided by the mode degree. For example, if there is a 100*%* probability that modes of type *D* = 50*%* exist, then there will be two modes that each contain half of the particles and E[50%]=2 .

The Expected entropy of a mode degree type *D* is the expected number of modes of that degree, multiplied with the entropy of modes of that degree. Since variance growth is identical across all modes every mode of a given degree will have identical entropy. To calculate the expected entropy of the system we sum over the expected entropy for each mode degree type giving

$$E\left[u_{k}\right]=\underset{d\in D}{\sum }P_{d,k}\frac{1}{d}\overset{H}{\underset{h=0}{\sum }}-d\varphi_{k}\left[b^{h}\right]\log\left(d\varphi_{k}\left[b^{h}\right]\right)$$ (46)

Which simplifies into Eq. 29. Lemma 5 provides the probability *P*_*D*_ of each mode type existing. The lower bound for the average entropy is then the average of Eq. 29 for *k* = [0, *T*_*Q*_], given by Eq. 27. QED.

This theorem represents the best case scenario using a perfect multi-agent path planner. In cases with sub-optimal path planners it is possible that more UAVs would be required to maintain the desired lower bound of average entropy.

While entropy is a useful metric it is not intuitive. To make Theorem 1 more intuitive we provide Corollary 1 as a means of transforming the lower bound of average entropy into the upper bound for the average variance in location certainty per target estimate. The variance in location certainty is the positional variance $\sigma_{p}^{2}$ in a single mode. For example if there are five modes each with a positional variance of 3 m then we know that the target is in one of five locations with a 3 m variance in each of the five estimates. This corollary makes the additional assumption that the number of modes in the particle filter when at the lower bound of average entropy is known. The positional variance $\sigma_{p}^{2}$ , number, and degree of modes are used when calculating entropy. Because of this, if the exact number and type of modes is unknown there is a many-to-many relationship between the entropy and the positional variance in the modes of the system. However, if the number of modes are known then there is a one-to-one relationship between entropy and the positional variance in each mode of the RBPF under Assumptions 1, 6, and 7\enleadertwodots

Corollary 1. *Given assumptions* 1*,* 6*, and* 7*. If the number of modes of each degree is* *D* *, then the upper bound on the average variance in target location certainty per estimate can be calculated by solving for* $\sigma_{p}^{2}$ *in*

$$B_{L}=\underset{d\in D}{\sum }\overset{J}{\underset{j=0}{\sum }}\frac{-1}{\sqrt{2\pi \sigma_{p}^{2}}}\exp\left(\frac{\mu -b^{j}}{2\sigma_{p}^{2}}\right)\log\left(\frac{D\exp\left(\frac{\mu -b^{j}}{2\sigma_{p}^{2}}\right)}{\sqrt{2\pi \sigma_{p}^{2}}}\right).$$ (47)

Proof.

If the number of modes of each degree type is known then *P*_*D*_ = 1 for each degree type in Eq. 29. The upper bound for the location certainty per target estimate is the positional variance of the RBPF when the entropy is equal to *B*_*L*_. To get the variance corresponding to *B*_*L*_, we must numerically solve Eq. 47 for $\sigma_{p}^{2}$ . QED.

As an example, consider the map shown in Figure 2 with ten targets. It is possible to determine the lower bound for entropy based on the number of UAVs deployed. With just one UAV the lower bound for entropy calculated in Eq. 27 is *B*_*L*_ = 2.89. If there is only one mode present of degree type *D* = 1 then we can find the upper bound on the target location certainty by solving Eq. 47 where *D* = 1. This shows that the upper bound on the target location certainty is 19 m. If this mission requires an upper bound of 16 m, we know that one UAV is insufficient. Setting $\sigma_{p}^{2}=16$ in Eq. 47 and solving for *B*_*L*_ we get the required lower bound on entropy to meet mission requirements. To get the number of UAVs required for the mission we solve for *Q* in Eq. 27. By rounding up to the nearest integer we find that a minimum of three UAVs are required to maintain the required average location certainty in target estimates.

## 6 Simulation Results

**FIGURE 9:**
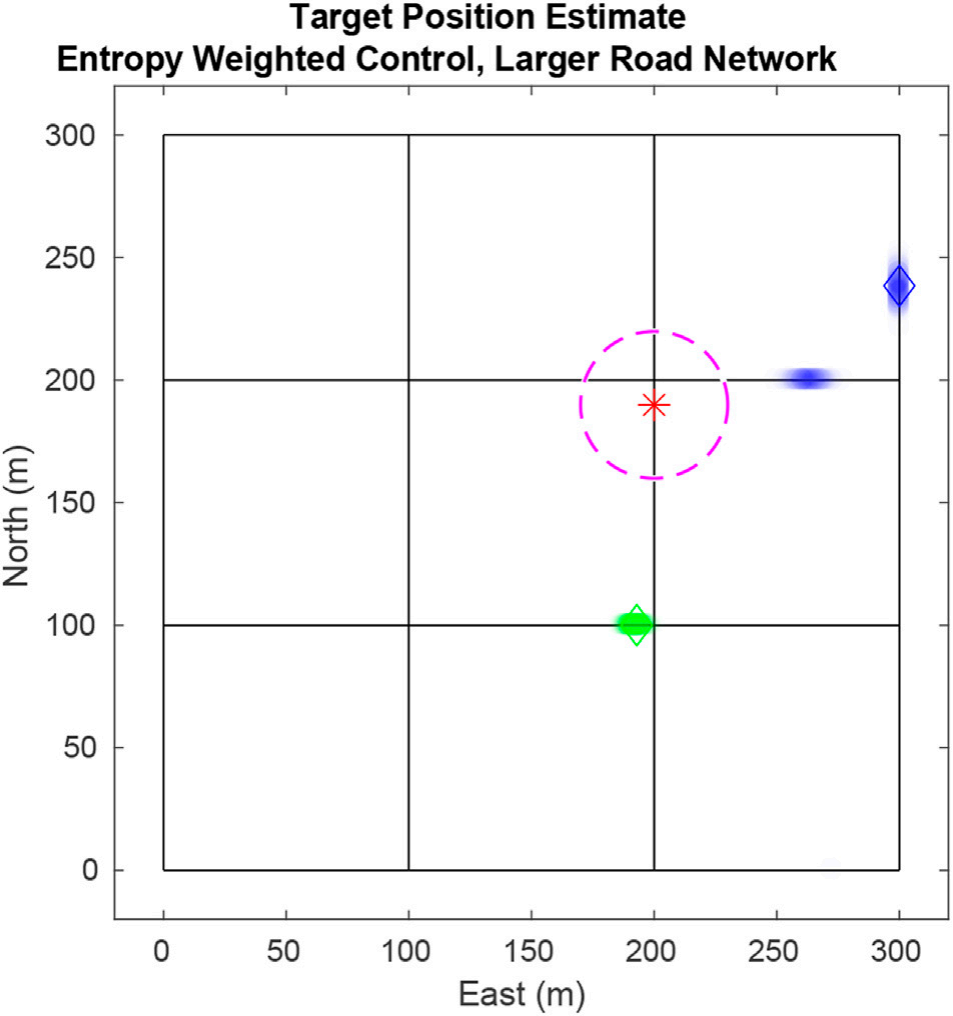
Simulation tracking two targets on a 3 × 3 city block map. Here the UAV is drawn toward the blue target because its estimate has higher entropy as seen by the two particle modes (blue dots).

In this section, simulation results are provided using the 3 × 3 road network in Figure 9 with two targets (blue and green diamonds), each travelling at a nominal 10 m per second. The UAV (red star) flies at 40 m per second (the speed of a small to medium-sized fixed-wing UAV) and has a fixed, downward looking sensor whose field-of-view is depicted by the magenta dashed circle. In the figure, particles are depticted with the transparent blue or green dots. In the top level of the filter, ten particles estimate data association histories. Each top level particle has one tracking PF per target (totalling 20 tracking filters) and each tracking PF has 500 particles. The target weighting sigmoid in Eq. 20 uses gain *a* = 10 and the measurement sensor noise is *R* = 5.

**FIGURE 10:**
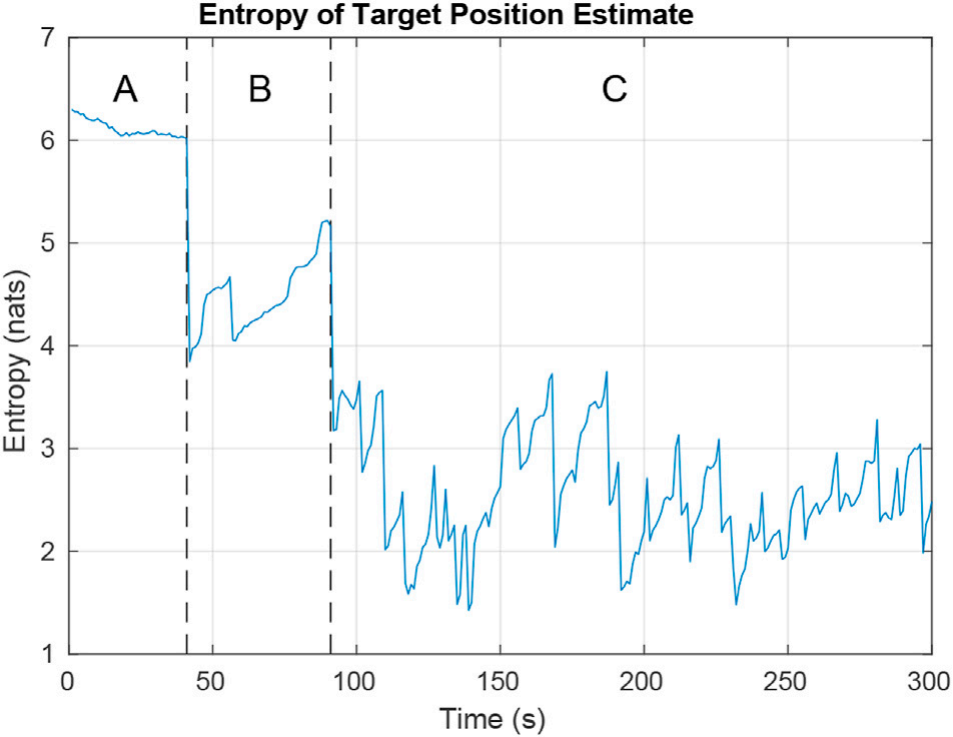
Entropy while simulating the tracking of two targets on a 3 × 3 city block map. In region **A**, the UAV is searching for targets. In region **B**, the UAV has located the first target and is looking for the second. In region **C**, the UAV is trying to minimize entropy across both targets.

Figure 10 shows how the combined entropy of the filter evolves as the UAV tries to find and follow both targets. In region A, the UAV has not found either target. The plot shows some decline in entropy as negative updates are applied and areas are ruled out. Region B shows the time after the first target has been found and priority switches to finding the second target. In Region C, the UAV tries to balance time between following each target to minimize total entropy. Rapid increases in entropy result when targets reach an intersection and hypotheses split. Steep declines in entropy result from positive measurements of the target and negative measurements ruling out hypotheses.

The simulation was run 1000 times using four different controllers and the entropy averaged over all the runs. The controllers compared were the Exhaustive RHC from Section 4.2, deep-RL planner from Section 4.3, an ideal planner with perfect knowledge of the target locations, and a stochastic planner that randomly decides paths whenever it encounters intersections.

**FIGURE 11:**
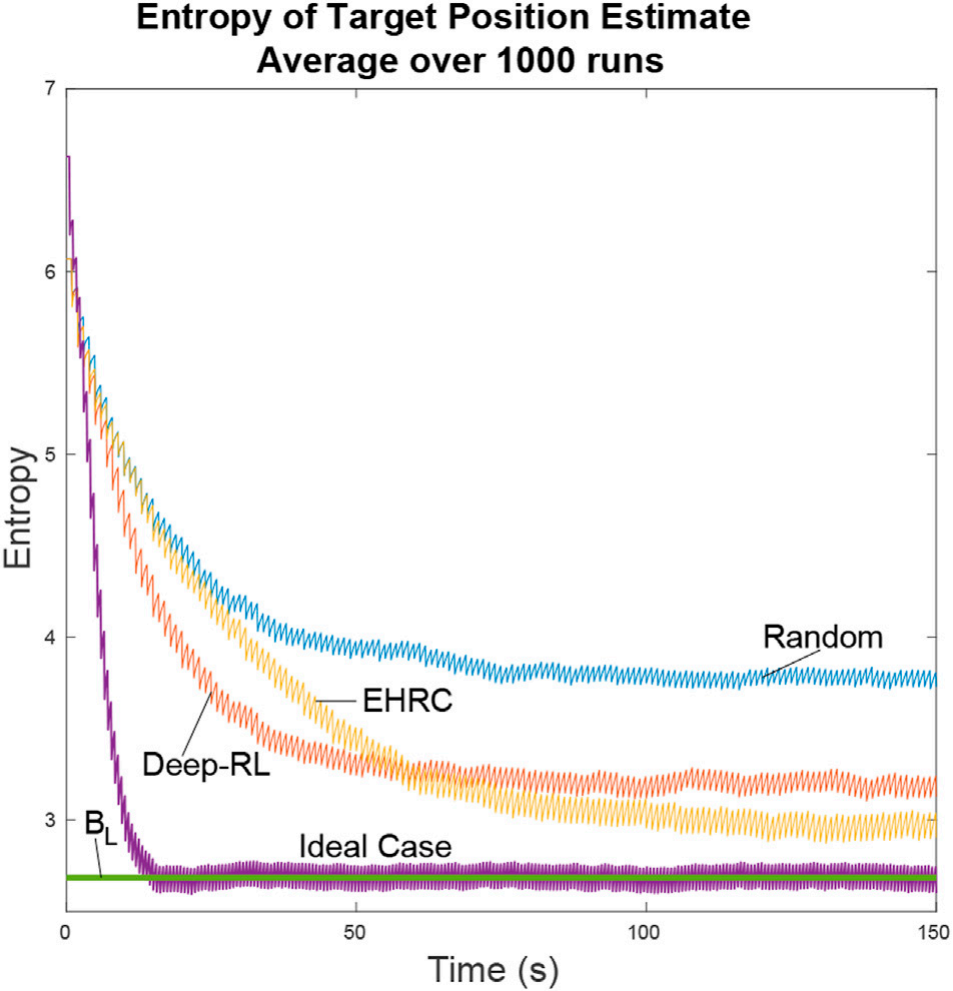
The ideal scenario and the lower bound are included for comparison. In the ideal scenario the UAV is always aware of the actual positions of the targets and flies directly between the two. The Lower Bound describes the average lower bound for entropy. We can see that the neural net path planner dropped in entropy faster than the exhaustive path planner. This is because the neural net learned efficient search patterns for uniformly distributed particles while the exhaustive path planner only made efficient decisions after it knew the general locations of the targets.

Figure 11 shows that the different path planners have varying degrees of success. The exhaustive RHC approaches the ideal case, the deep-RL planner coming in a close second, and the random path planner falls far behind. Considering that the ideal case is the best possible value, the deep-RL result approximately halves the entropy in the particle filter in comparison with the random path planner. While when using the exhaustive RHC path planner, the entropy is cut to almost 1/3.

The lower bound line is a result of the lower bound entropy algorithm described in Section 4 that tells us how well the UAVs would perform under ideal conditions. In ideal conditions whenever a UAV has to guess as to which location is most likely to have the target the UAV makes the right choice. As a result we can see the ideal case quickly converges onto the lower bound of entropy. All other path planners will minimize the entropy of the system as best they can, but lacking perfect knowledge they will come short.

The Exhaustive RHC yields the next best results over time as can be expected when doing an exhaustive path analysis. Where this planner falls short is during the initial search for the targets. When particles are distributed uniformly over the map the exhaustive path planner is forced to make random decisions. In comparison, the neural net initially does better by learning an efficient search pattern. In both of these cases the UAV has limited information about the target location and so cannot do as well as the ideal planner.

The other main limitation of both these path planners is their inability to scale well. The exhaustive planner’s runtime execution is *O*(*MNd* ^*L*^) meaning that with a large enough complex map the exhaustive planner will fail to execute in real time. Conversely, regardless of map size the neural net is always *O*(1). However, the neural net training time scales poorly with increasing map sizes. One of the ways the exhaustive planner could be modified to handle large maps would be to take advantage of simulated annealing (Miao and Tian (2013)) to reduce the search space required by a large map.

Many other path planners, such as jump point search (Harabor and Grastien (2014)) or Rollout Policy (Tian et al. (2008)), provide alternative efficient ways to plan through large environments, each with their own advantages and disadvantages. It is also possible that modifications to the neural net could reduce the training time to an acceptable level for larger maps. As a proof of concept however, this shows that the Deep-RL planner is a competitive alternative.

## 7 Hardware Results

**FIGURE 12:**
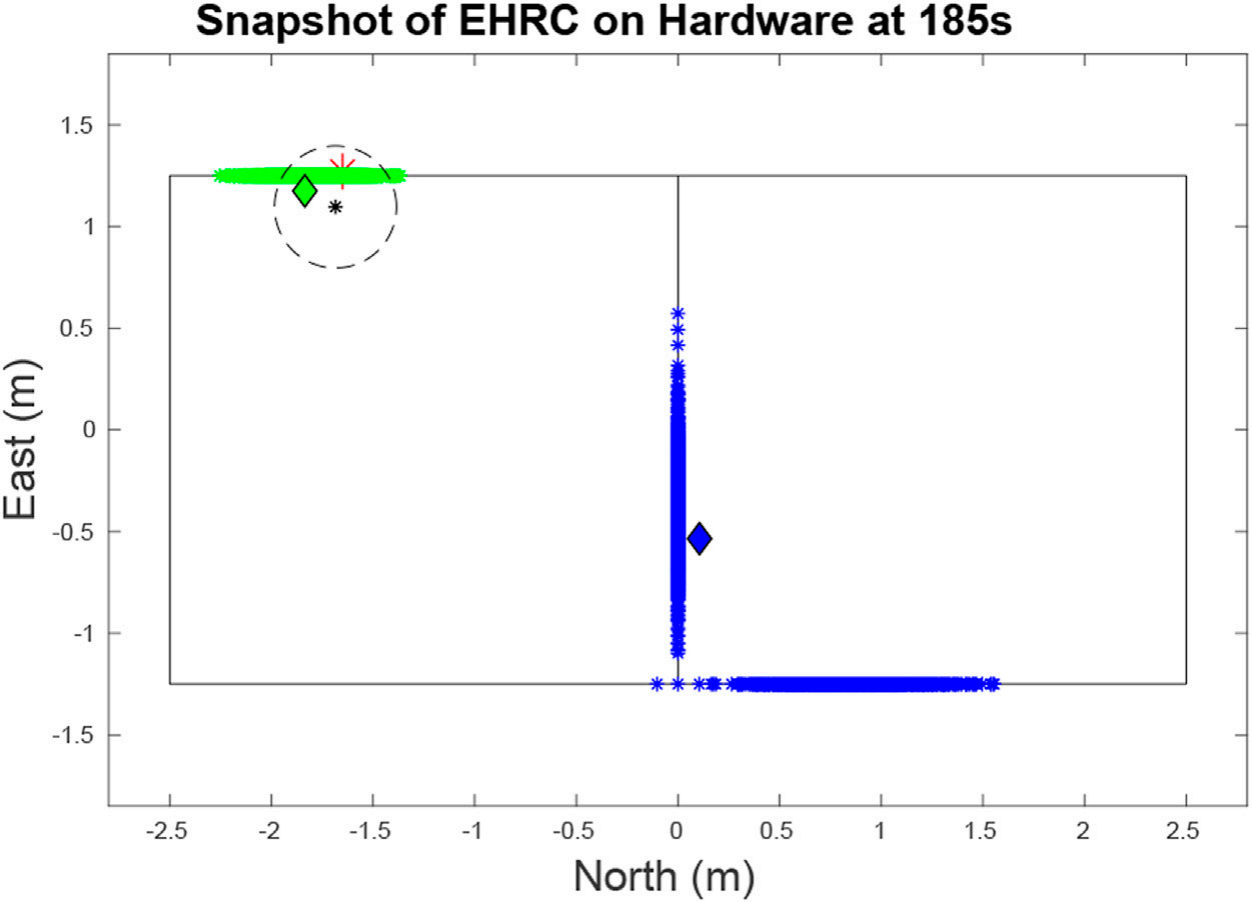
Snapshot at at 185 s of the target and UAV states during a hardware test. The red asterisk represents the quadcopter. The green and blue diamonds are the current locations of each of the two vehicles. As can be seen, the green particle cloud is closely aligned with the actual position of its target. And while there are multiple modes for the blue target representing different estimates, the blue target is closely aligned with one of its modes. The black star and circle represents the estimate and error margin of a target sighting, which when funneled through our data association algorithm, performs a positive update on the green target.

**FIGURE 13:**
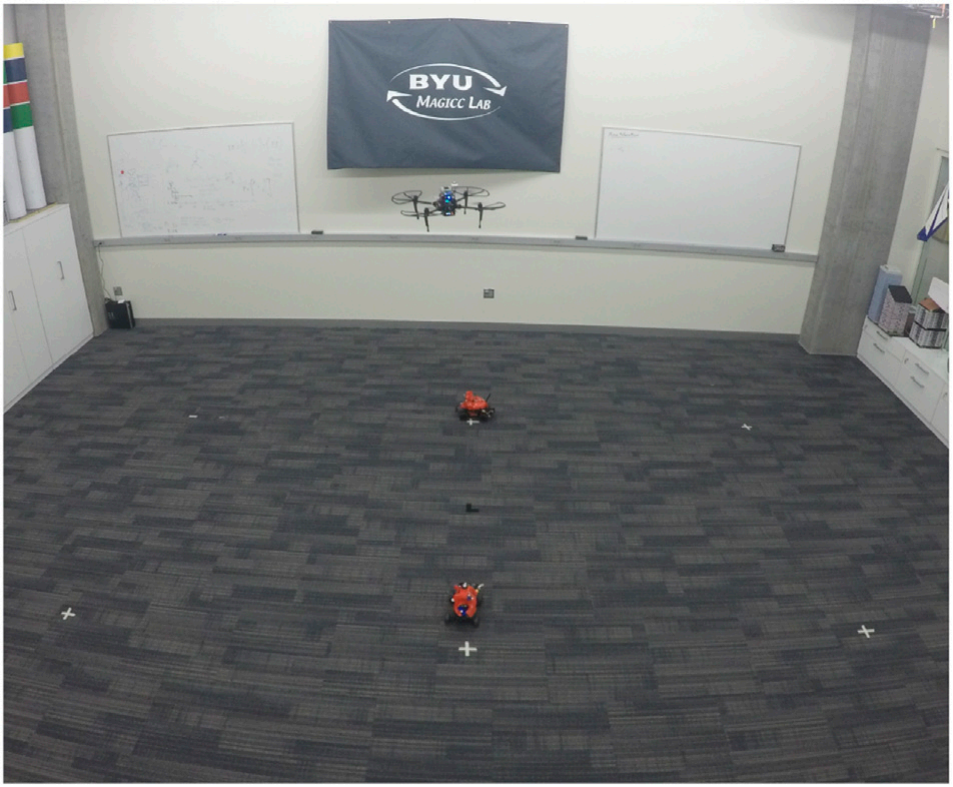
A photo of our hardware in action. The quadcopter is hovering above the second target while it is still unaware of the location of the first target. The targets are small red RC cars running dubins path between the six markers on the ground.

We used hardware to verify the efficacy of both the RBPF and the lower entropy bound algorithm. Flight tests were performed in a motion capture room using the road map shown in Figure 12. We used a quad-copter equipped with an ocam camera as the UAV. The onboard computer was a TX2 which handled the vision processing and the autopilot. The quadcopter used a PID controller with waypoint following and near constant velocity was managed by saturating the error in the PID loop at 0.3 m. A photo of the hardware in flight is included in Figure 13.

Target detection was handled by inputing the camera data into Visual MTT (Ingersoll (2015)) with RRANSAC (Niedfeldt and Beard (2014)). Visual MTT used the color detector to identified the target and R-RANSAC outputed the track information in camera frame coordinates. Assuming a flat earth model we transformed the camera frame coordinates into the inertial frame. The camera feed had a latency of approximately 0.4 s which needed to be accounted for when doing the transformation into the inertial frame. By accounting for this latency issue we were able to maintain an error less than half a meter on average for our estimate.

**FIGURE 14:**
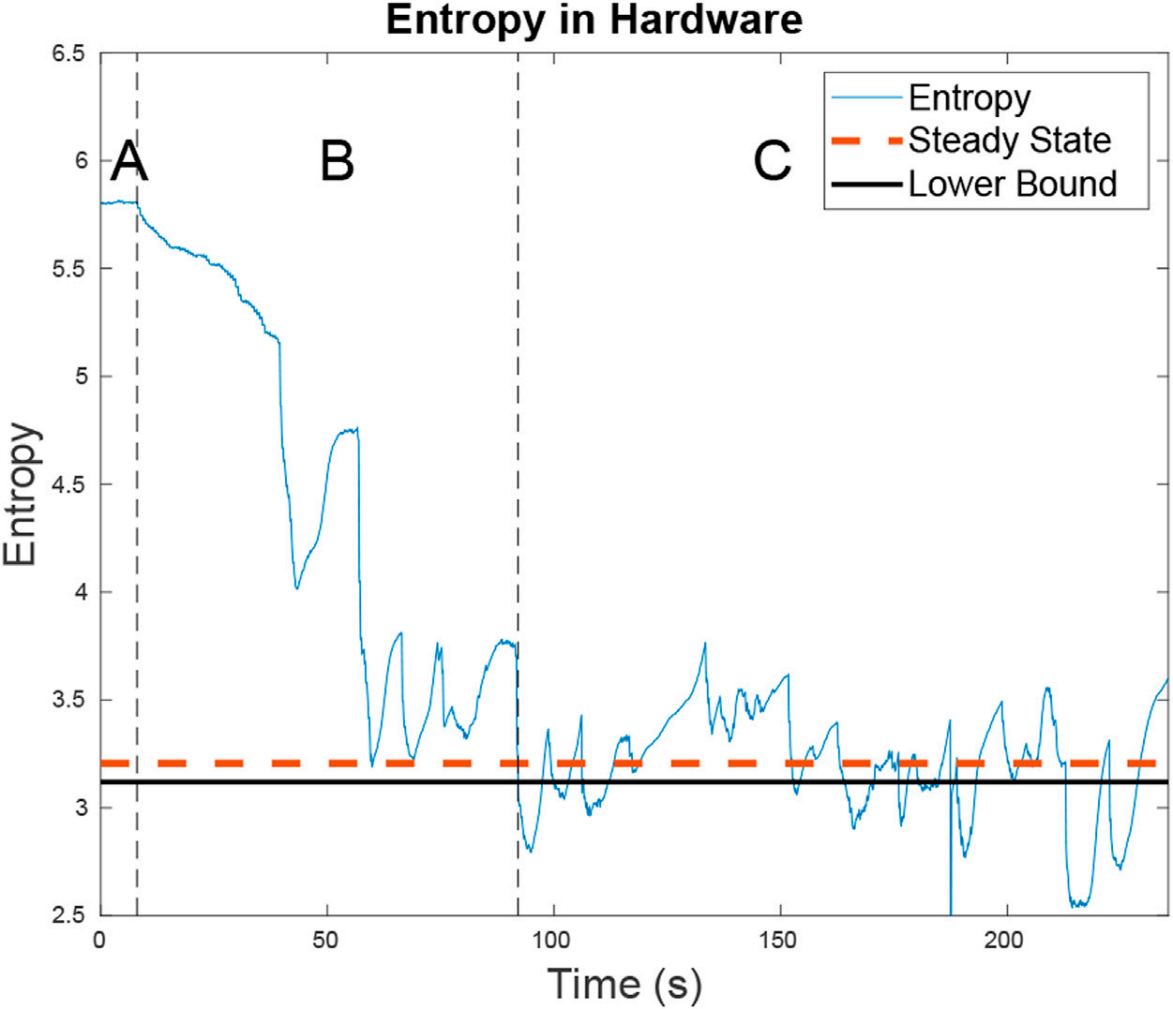
The entropy for the two targets as well as the steady state entropy for the hardware test is shown. In region **A**, the UAV is searching for targets. In region **B**, the UAV has located the first target and is looking for the second. In region **C**, the UAV is trying to minimize entropy across both targets. The steady state entropy of around 3.206 which is just slightly higher than the lower bound of 3.119 U of entropy. This is expected as on a small map the ERHC planner will work comparatively to the ideal planner.

The targets are two small RC cars executing Dubins paths (Beard and McLain (2012)) on a road network with PID control. Even with the large turn radius of the cars and their inability to strictly adhere to the road network as well as a large variance in their speed control, the RBPF accurately estimates the car movement using the parameters in Table 1. The cars, which move with near constant velocity, chose random paths when encountering intersections and used Dubins paths to minimize their deviations from the road network. The vehicles travelled at an average speed of 0.24 m/s and the quadcopter maintained an average speed of approximately 1.25 m/s using the Exhaustive RHC from Subsection 4.2 with the parameters in Table 1. As seen by Figure 14 the average entropy was 3.39. On this map the target certainty was on average, within a variance of 1 m.

**TABLE 1:** Parameters used by the Path Planner and the RBPF to maximize target certainty given our hardware costraints.

Path planner	
*δ*_*t*_	2
discount	0.8
Lookahead Max	3
Waypoint Threshold	0.15
**RBPF**	
Target Velocity	0.24
Target *σ*_*v*_	0.2
Max Targets	2
R	0.3
*P*_*FA*_	0.1
*P*_*null*_	0.25
*δ*_*t*_	0.15
Top Level Particles	10
*N*	1000

## 8 Conclusion

By employing a Rao-Blackwellized Particle Filter, we have shown that data association can be performed effectively, even when the target leaves and re-enters the sensor’s field-of-view. We then proved a theorem for calculating the upper-bound for average target location certainty based on the number of UAVs available.

Further, we have shown that a neural net trained using deep reinforcement learning is capable of learning efficient map sweeping strategies when target locations are unknown, after the target locations are known it reliably tracks the targets. After the initial search, an exhaustive RHC is more efficient at tracking targets than the neural net. However, it requires significantly more computations. Both the exhaustive receding horizon controller and deep-RL controllers significantly improve tracking performance and target location certainty compared with a random search pattern.

Future work includes employing a higher fidelity motion model for the targets, such as that of (Cheng and Singh (2007)). The RBPF filter could also be augmented to handle an unknown number of targets similar to the technique used in (Thrun et al. (2006)) for estimating the number of landmarks in SLAM. In addition, our deep-RL path planner could be enhanced by taking advantage of advances in transfer learning that would allow the neural net to work on multiple maps with a minimal increase in training time (Banerjee and Stone (2007)). Finally, the lower bound entropy algorithm could also be augmented to account for mode merging when looking at long periods between target sightings. This would allow the algorithm to handle increasingly complex road networks.

## Data Availability

The original contributions presented in the study are publicly available. This data can be found here: https://github.com/erconui/mass.git.
